# Effect of Leukocyte‐Platelet‐Rich Fibrin on Peri‐Implant Keratinized Mucosal Width and Mucosal Thickness Following Immediate Implant Placement: A Randomized Clinical Trial

**DOI:** 10.1155/ijod/5014584

**Published:** 2026-11-03

**Authors:** Najmehsadat Valedsaravi, Mehrshad Ahmadi Dizgah, Amirhossein Moaddabi, Abolfazl Hosseinnataj

**Affiliations:** ^1^ Department of Periodontics Faculty of Dentistry Mazandaran University of Medical Sciences Sari Iran mazums.ac.ir; ^2^ Department of Oral and Maxillofacial Surgery Faculty of Dentistry Tehran University of Medical Sciences Tehran Iran tums.ac.ir; ^3^ Department of Oral and Maxillofacial Surgery Faculty of Dentistry Mazandaran University of Medical Sciences Sari Iran mazums.ac.ir; ^4^ Department of Biostatistics Faculty of Health Mazandaran University of Medical Sciences Sari Iran mazums.ac.ir

**Keywords:** immediate implant placement, keratinized mucosa, keratinized mucosal width, leukocyte-platelet-rich fibrin, peri-implant mucosal thickness

## Abstract

**Background:**

Adequate keratinized peri‐implant soft tissue supports plaque control, patient comfort, and long‐term tissue stability. This study evaluated whether application of a leukocyte‐platelet‐rich fibrin (L‐PRF) plug during immediate implant placement improves keratinized mucosal width (KMW) and peri‐implant mucosal thickness (PMT).

**Materials and Methods:**

In this parallel‐group randomized clinical trial, 60 systemically healthy patients requiring extraction and immediate implant placement were allocated to an L‐PRF group or a control group. The primary outcome was KMW at final follow‐up, whereas PMT at final follow‐up was considered a secondary clinical outcome. Both outcomes were analyzed using baseline‐adjusted ANCOVA.

**Results:**

All randomized participants completed the study. At baseline, PMT did not differ significantly between groups (*p* = 0.505), whereas KMW was greater in the L‐PRF group (*p* = 0.019). At the final follow‐up, both KMW and PMT increased significantly in both groups (*p*  < 0.001). The L‐PRF group demonstrated greater PMT and KMW at the final follow‐up than the control group (*p*  < 0.001 for both comparisons). After adjustment, group allocation remained a significant predictor of both PMT (*p*  < 0.001) and KMW (*p*  < 0.001). No adverse events were reported.

**Conclusion:**

In this trial, application of an L‐PRF plug during immediate implant placement was associated with greater KMW and PMT at final follow‐up than standard surgical closure alone. These findings should be interpreted with caution given the baseline KMW imbalance and the lack of consistently recorded surgical and site‐related variables.

**Trial Registration:** Iranian Registry of Clinical Trials: IRCT20221228056958N2

## 1. Introduction

Tooth loss due to trauma, extensive caries, or periodontal disease can compromise mastication and dental esthetics and reduce the quality of life [1–3]. Dental implants are widely used for replacement of missing teeth, with high long‐term survival rates when biological and prosthetic principles are respected [4]. Immediate implant placement after extraction is attractive because it can reduce treatment time and surgical sessions and may improve patient acceptance [5–7].

The presence of an adequate band of keratinized mucosa around dental implants is essential for achieving optimal esthetic outcomes, establishing an effective peri‐implant soft tissue seal, facilitating oral hygiene, and ensuring long‐term implant success [8]. Healthy peri‐implant soft tissue contributes to the formation and maintenance of a stable biologic width and protects the underlying peri‐implant structures. Accordingly, the role of the peri‐implant soft tissue seal, biologic width, and keratinized mucosal width (KMW), as well as the importance of effective plaque control in preserving peri‐implant tissue health, has become a major focus of contemporary implant research [8]. Insufficient keratinized mucosa, particularly widths of less than 2 mm, has been associated with increased plaque accumulation, peri‐implant soft tissue inflammation, mucosal recession, marginal bone loss, a higher prevalence of peri‐implantitis, and greater patient discomfort [9].

Autogenous grafts have been used to increase KMW around dental implants and have been associated with favorable clinical outcomes, including improved bleeding indices and enhanced marginal bone levels [10]. More recently, platelet‐rich fibrin (PRF) has been introduced as an autologous, simple, and cost‐effective regenerative biomaterial with promising results in preparing implant sites and supporting peri‐implant tissue healing [11]. The primary biological advantage of leukocyte‐PRF (L‐PRF) is the sustained release of concentrated growth factors at the surgical site, which may enhance the wound healing process [12]. PRF is a biocompatible material that has demonstrated stimulatory effects on soft‐ and hard‐tissue regeneration, angiogenesis, and immune modulation [13].

Despite growing clinical evidence supporting the beneficial effects of PRF in implant dentistry [14], the number of studies specifically evaluating the efficacy of L‐PRF remains limited, and controversy persists regarding its routine use in dental implant therapy [15]. Therefore, the present study aimed to evaluate the effect of an L‐PRF plug on KMW and peri‐implant mucosal thickness (PMT) following immediate dental implant placement. The null hypothesis was that the application of an L‐PRF plug would have no significant effect on KMW or PMT after immediate implant placement.

## 2. Materials and Methods

### 2.1. Study Design and Registration

This parallel‐group randomized controlled clinical trial was conducted at the Specialty Clinic of the School of Dentistry, Mazandaran University of Medical Sciences (Sari, Iran). This study was approved by the Ethics Committee of the Mazandaran University of Medical Sciences, Sari, Iran, under the Approval Code IR.MAZUMS.REC.1402.618. Written informed consent was obtained from all participants before enrollment. The study was reported in accordance with the CONSORT guidelines for randomized clinical trials. A completed CONSORT checklist was prepared and submitted as separate files.

#### 2.1.1. Participants, Eligibility Criteria, and Settings

Systemically healthy patients (ASA I) who required tooth extraction and planned immediate implant placement were screened for eligibility. Inclusion criteria included the absence of untreated periodontal disease [16], good oral hygiene, no active infection, and adequate patient cooperation. Eligible participants were required to present with a tooth indicated for extraction due to irreparable caries, hopeless periodontal prognosis, or crown or root fracture and to be willing to attend all subsequent treatment sessions and follow‐up visits [17].

The exclusion criteria were pregnancy or nursing, psychological problems, pathologies at the surgical site [16], allergy to medications used in the study, traumatic occlusion, malocclusion, systemic conditions interfering with wound healing [17], history of coagulopathies, intake of anticoagulants, immunocompromised status, debilitating systemic diseases [12], smoking more than 10 cigarettes/day, and intake of medications affecting the wound healing process. Patients who did not attend follow‐up visits, were unwilling to continue participation, used antibiotics, anti‐inflammatory drugs, or other medications affecting wound healing outside the standardized postoperative protocol, had defective extraction sockets or periapical lesions, or required extensive bone grafting procedures such as guided bone regeneration or bone block grafting were excluded.

Participants were withdrawn from the study during follow‐up for postoperative infection or healing complications, the need for medications affecting soft‐ or hard‐tissue healing, poor oral hygiene, failed osseointegration, or unwillingness to continue participation.

Participant recruitment was conducted over 12 months (2022–2023) at the Specialty Clinic of Mazandaran University of Medical Sciences, and all participants completed follow‐up through the final assessment, ~4 months plus 2 weeks after implant placement.

### 2.2. Sample Size Calculation

The sample size was calculated using the change in the buccal KMW as the primary basis for estimation. Because limited prior randomized evidence was available on L‐PRF use for peri‐implant keratinized mucosal outcomes after immediate implant placement, the study by Zhang et al. [18], which evaluated PRF in post‐extraction healing, was used as the closest available reference to estimate the expected magnitude and variability of soft‐tissue dimensional change. Based on the reported mean changes of 1.05 ± 0.78 mm and 2.08 ± 1.67 mm, an absolute between‐group difference of ~1.0 mm was considered clinically relevant. Using a two‐sided alpha level of 0.05 and 80% power, the required sample size was estimated to be 27 participants per group. To compensate for an anticipated dropout rate of ~10%, the final sample size was increased to 30 participants per group for a total of 60 participants.

### 2.3. Randomization and Blinding

The patients were randomly assigned to two groups using block randomization with blocks of 4. For this purpose, patients who were found to be eligible for study inclusion according to their panoramic radiograph and cone‐beam computed tomography scan received a code from 1 to 60.

Group allocation was performed using block randomization generated by Random Allocation 2 software. Allocation concealment was ensured using sequentially numbered, sealed, opaque envelopes prepared by an independent staff member not involved in patient recruitment or treatment. Upon patient enrollment, the surgeon opened the envelope immediately before the procedure, and the assigned intervention was performed accordingly.

The study had a single‐masked design. The examiner responsible for the outcome assessment was blinded to group allocation. Blinding the surgeon was not feasible given the nature of the intervention.

#### 2.3.1. Interventions

Written informed consent was obtained from all participants. A single experienced periodontist performed all surgical procedures to ensure operator consistency. Preoperative CBCT was used for implant planning, evaluation of the extraction site, and confirmation of eligibility. Radiographic assessment was used to evaluate the surgical site and to exclude cases with periapical lesions, defective extraction sockets, or the need for extensive bone grafting procedures, such as guided bone regeneration or bone block grafting.

Local anesthesia was administered using 2% lidocaine with 1:80,000 epinephrine (Exir Pharmaceuticals, Iran). Teeth were extracted atraumatically using a periotome, elevator, and forceps. Osteotomy preparation followed the manufacturer’s protocol (Surgic, NSK), and the implants were inserted with a torque of 35 N·cm. A primary stability of at least 15 N·cm was achieved by extending drilling 3 mm beyond the apical bone or by engaging the lateral socket walls.

A two‐piece sandblasted, large‐grit, acid‐etched (SLA) titanium implant (Dentis or Snucone; Korea) was placed immediately after extraction, positioning the implant platform 1 mm apical to the buccal bone crest. In cases where a horizontal gap exceeding 2 mm was present between the implant surface and the socket walls, the defect was grafted using a combination of allograft (MBA, Regen) and xenograft materials (Bone + B; Arpamed).

In the control group, no additional intervention was performed following implant placement, and the flap was coronally repositioned to prevent implant exposure. In the intervention group, an L‐PRF plug was placed over the implant immediately after insertion (Figure 1).

**Figure 1:**
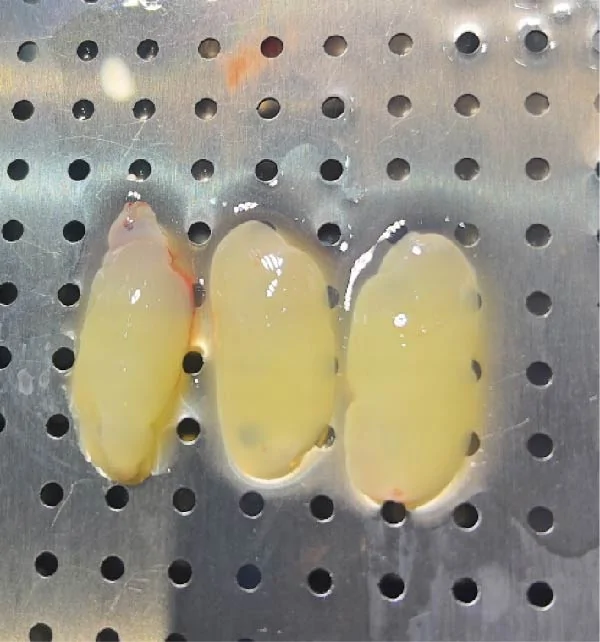
An L‐PRF plug was prepared for placement over the implant in the intervention group.

L‐PRF was prepared by collecting 9 mL of venous blood from each patient into sterile plain glass tubes without an anticoagulant. Centrifugation was initiated immediately after blood collection using an IntraSpin centrifuge (Intra‐Lock, USA) at 2700 rpm (408 g) for 12 min. After centrifugation, the fibrin clot was separated from the red blood cell layer and gently compressed with sterile gauze to prepare an L‐PRF plug. The prepared L‐PRF plug was placed over the implant in the intervention group before flap closure.

All participants received the same standardized postoperative medication protocol, including amoxicillin 500 mg every 8 h, ibuprofen 400 mg every 6 h, and 0.2% chlorhexidine mouthwash (Iran Naju Pharmaceutical Co., Tehran, Iran) three times daily for 1 week.

Flap management and closure were standardized in both groups. The same flap design, flap advancement, coronal repositioning, and suturing protocol were used for all participants. In both groups, the flap was coronally repositioned to prevent implant exposure and achieve passive closure, and the surgical site was closed with 6/0 nylon sutures. The only difference between the two groups was that an L‐PRF plug was placed over the implant in the intervention group before flap closure.

#### 2.3.2. Outcomes

The primary outcome was buccal KMW at the final follow‐up, analyzed with adjustment for baseline KMW. The secondary clinical outcome was PMT at the final follow‐up, analyzed with adjustment for baseline PMT. Baseline measurements were obtained immediately before tooth extraction, and follow‐up measurements were obtained at the corresponding implant site after healing.

KMW was measured at the mid‐buccal aspect of the tooth/implant site as the distance from the mucosal margin to the mucogingival junction. The mucogingival junction was identified using the rolling technique (Figure 2), and measurements were performed using a periodontal probe [16].

**Figure 2:**
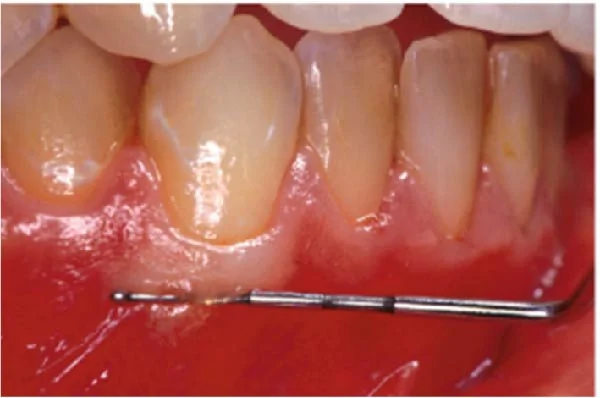
Measurement of buccal keratinized mucosal width at the mid‐buccal aspect using the rolling technique to identify the mucogingival junction.

PMT was assessed by transgingival bone sounding with a Williams periodontal probe after local anesthesia (Figure 3). The measurement was performed perpendicular to the mucosal surface at a standardized mid‐buccal point of the tooth/implant site until bone contact was detected.

**Figure 3:**
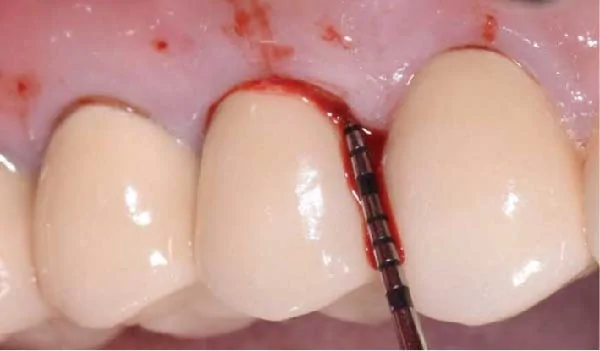
Assessment of peri‐implant mucosal thickness by transgingival bone sounding at a standardized mid‐buccal point, perpendicular to the mucosal surface.

All measurements were performed by the same examiner, who was blinded to the group allocation. To reduce measurement error, each measurement was repeated twice, and the mean value was used for statistical analysis. Before data collection, the examiner was calibrated using 50 pilot cases. Intra‐examiner reproducibility was assessed by repeating measurements in 20% of randomly selected cases after 1 week, yielding intraclass correlation coefficients of 0.817 for KMW and 0.875 for PMT.

Baseline KMW and PMT measurements were obtained immediately before tooth extraction. Four months after immediate implant placement, reentry surgery was performed, and the healing abutment was placed. Final KMW and PMT measurements were obtained 2 weeks after healing abutment placement, corresponding to ~4 months plus 2 weeks after implant placement.

#### 2.3.3. Follow‐Up

Patients were clinically monitored at 1 week, 1 month, 3 months, and 4 months after implant placement to assess postoperative healing and implant stability. Reentry surgery and healing abutment placement were performed at ~4 months, and final KMW and PMT measurements were obtained 2 weeks later. The same surgeon conducted all follow‐up visits.

#### 2.3.4. Statistical Analysis

Statistical analyses were performed using SPSS Version 22.0 (SPSS Inc., Chicago, IL, USA) with a significance level of 0.05. Continuous variables were assessed for normality using the Shapiro–Wilk test and visual inspection of histograms and *Q*–*Q* plots. Homogeneity of variance was assessed using Levene’s test. Normally distributed continuous variables were reported as the mean ± standard deviation.

Between‐group comparisons were performed using independent‐samples *t*‐tests, and within‐group comparisons between baseline and final follow‐up were performed using paired‐samples *t*‐tests. Categorical variables were compared using the chi‐square test. ANCOVA was used to compare final follow‐up KMW and PMT values between groups after adjustment for baseline outcome values, age, and sex.

KMW was prespecified as the primary outcome, whereas PMT was considered a secondary clinical outcome. Accordingly, no multiplicity adjustment was required for the primary outcome analysis. The PMT findings and any other secondary or exploratory comparisons should be interpreted cautiously.

Additional socket‐, graft‐, implant system‐, and implant site‐related variables were not included in the adjusted models because these data were not consistently available for all participants. This limitation was considered when interpreting the findings. All randomized participants were included in the final analysis in accordance with the intention‐to‐treat principle. No protocol deviations requiring per‐protocol analysis occurred.

## 3. Results

### 3.1. Participant Flow

Sixty eligible participants who met the inclusion criteria and provided written informed consent were randomized, with 30 allocated to the L‐PRF group and 30 allocated to the control group. All allocated participants received the assigned intervention. No participants were lost to follow‐up, and all randomized participants were included in the intention‐to‐treat analysis. Participant flow from randomization to analysis is presented in Figure 4.

**Figure 4:**
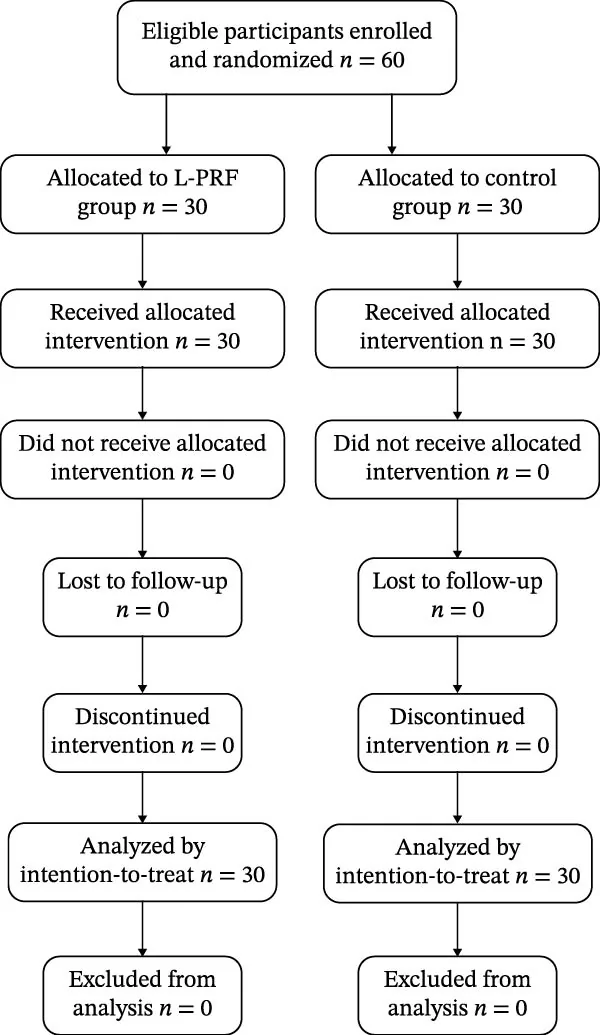
Participant flow diagram from randomization to analysis. Complete prerandomization screening data, including the total number assessed for eligibility and detailed reasons for exclusion, were not consistently available from the study records. L‐PRF, leukocyte‐platelet‐rich fibrin.

### 3.2. Baseline Characteristics

Of the 60 participants, 36 (60%) were male and 24 (40%) were female. The intervention group comprised 19 males (63.3%) and 11 females (36.7%), while the control group included 17 males (56.7%) and 13 females (43.3%). There was no significant difference in gender distribution between the groups (*p* = 0.598).

The mean age of the participants was 39.57 ± 10.65 years (range: 18–60 years). Mean age was 39.87 ± 11.04 years in the L‐PRF group and 39.27 ± 10.43 years in the control group, with no significant difference between groups (*p* = 0.830).

### 3.3. Clinical Outcomes

#### 3.3.1. Primary Outcome: KMW

At baseline, mean KMW was significantly higher in the L‐PRF group than in the control group (*p* = 0.019). At final follow‐up, ~4 months plus 2 weeks after implant placement, KMW remained significantly greater in the L‐PRF group than in the control group (*p*  < 0.001) (Table 1). Both groups demonstrated a significant increase in KMW from baseline to follow‐up (*p*  < 0.001 for both).

**Table 1:** Keratinized mucosal width (mm) in the two groups at baseline and final follow‐up after dental implant placement.

Time	L‐PRF	Control	*p* Value^a^
Baseline	3.87 ± 1.26	3.17 ± 0.98	0.019
Final follow‐up	4.95 ± 0.94	3.60 ± 0.91	<0.001
*p* Value^b^	<0.001	<0.001	—

*Note:* Values are presented as mean ± standard deviation.

Abbreviation: L‐PRF, leukocyte‐platelet‐rich fibrin.

^a^Independent *t*‐test.

^b^Paired *t*‐test.

Because baseline KMW was significantly greater in the L‐PRF group, the final follow‐up comparison was performed using baseline‐adjusted ANCOVA. After adjustment for baseline KMW, age, and sex, group allocation remained significantly associated with the final follow‐up KMW. The adjusted mean KMW was 0.84 mm greater in the L‐PRF group than in the control group (95% CI: 0.60–1.09; *p*  < 0.001), whereas age and sex were not significantly associated with KMW (Table 2). Nevertheless, the baseline imbalance should be considered when interpreting the magnitude of the observed between‐group difference.

**Table 2:** Effects of variables on KMW using ANCOVA.

Variable		Regression coefficient	SE	95% CI	*p* Value
Gender	Female	0.02	0.12	−0.22, 0.27	0.857
	Male	—	—	—	—
Age		0.002	0.01	−0.01, 0.01	0.719
Group	L‐PRF	0.84	0.12	0.60, 1.09	<0.001
	Control	—	—	—	—
Baseline KMW		0.72	0.05	0.62, 0.83	<0.001

Abbreviations: CI, confidence interval; KMW, keratinized mucosal width; L‐PRF, leukocyte‐platelet‐rich fibrin; SE, standard error.

#### 3.3.2. Secondary Clinical Outcome: PMT

At baseline, no significant difference in mean PMT was observed between the L‐PRF and control groups (*p* = 0.505). At final follow‐up, ~4 months plus 2 weeks after implant placement, mean PMT was significantly greater in the L‐PRF group than in the control group (*p*  < 0.001) (Table 3). Both groups showed a significant increase in PMT from baseline to follow‐up (*p*  < 0.001 for both).

**Table 3:** Peri‐implant mucosal thickness (mm) in the two groups at baseline and final follow‐up after dental implant placement.

Time	L‐PRF	Control	*p* Value^a^
Baseline	2.60 ± 1.36	2.40 ± 0.90	0.505
Final follow‐up	3.50 ± 0.19	2.82 ± 0.80	<0.001
*p* Value^b^	<0.001	<0.001	—

*Note:* Values are presented as mean ± standard deviation.

Abbreviation: L‐PRF, leukocyte‐platelet‐rich fibrin.

^a^Independent *t*‐test.

^b^Paired *t*‐test.

After adjustment for baseline PMT, age, and gender using ANCOVA, group allocation remained a significant predictor of PMT. The adjusted mean PMT in the L‐PRF group was 0.55 mm greater than that in the control group (95% CI: 0.27–0.82; *p*  < 0.001). Age and gender were not significantly associated with PMT (Table 4).

**Table 4:** Effects of variables on PMT using ANCOVA.

Variable		Regression coefficient	SE	95% CI	*p* Value
Gender	Female	0.20	0.15	−0.09, 0.50	0.171
	Male	—	—	—	—
Age		−0.01	0.01	−0.02, 0.01	0.388
Group	L‐PRF	0.55	0.14	0.27, 0.82	<0.001
	Control	—	—	—	—
Baseline PMT		0.77	0.06	0.65, 0.90	<0.001

Abbreviations: CI, confidence interval; L‐PRF, leukocyte‐platelet‐rich fibrin; PMT, peri‐implant mucosal thickness; SE, standard error.

#### 3.3.3. Harms

No adverse events, complications, or treatment‐related side effects were reported in either group during the study period.

## 4. Discussion

This randomized clinical trial investigated the effect of applying an L‐PRF plug during immediate implant placement on the peri‐implant KMW and PMT. Although postoperative increases in peri‐implant mucosal dimensions were observed in both groups, L‐PRF use was associated with greater short‐term gains in KMW and PMT at the final follow‐up, ~4 months plus 2 weeks after implant placement.

The increase in PMT observed in the L‐PRF group may be clinically relevant as thicker peri‐implant soft tissue has been associated with improved tissue resilience, reduced susceptibility to recession, and enhanced long‐term stability. Similarly, a wider band of keratinized mucosa may facilitate plaque control, reduce peri‐implant inflammation, and improve patient comfort during oral hygiene procedures. These findings are biologically plausible and may be explained by the sustained release of growth factors, promotion of angiogenesis, and modulation of local inflammatory responses associated with L‐PRF.

Beyond sustained growth factor release, PRF may also exert part of its biological activity through extracellular vesicle‐mediated paracrine mechanisms. PRF‐derived extracellular vesicles have been suggested to contribute to immune regulation, fibroblast migration, angiogenesis, osteogenic responses, and early tissue healing [19]. This mechanism may partly explain the favorable short‐term peri‐implant mucosal changes observed in the L‐PRF group. However, the present clinical study did not directly evaluate extracellular vesicle activity.

A significant baseline imbalance in KMW was observed despite randomization, with higher baseline values in the L‐PRF group. Although baseline‐adjusted ANCOVA was used and group allocation remained significantly associated with follow‐up KMW, this adjustment cannot fully eliminate the interpretative limitation introduced by baseline imbalance. Therefore, the observed difference in KMW should be interpreted with caution as part of it may reflect more favorable baseline soft‐tissue conditions in the L‐PRF group.

The present findings are partly consistent with previous studies evaluating PRF‐based biomaterials in implant dentistry. Some investigations have reported limited or no benefit of L‐PRF for hard‐tissue outcomes or patient‐reported measures [20, 21]. These studies primarily focused on bone preservation or implant stability rather than on peri‐implant soft tissue dimensions. Differences in study design, outcome selection, follow‐up duration, and measurement methods may account for the heterogeneity in the reported results. In contrast, other clinical trials and case reports have demonstrated favorable effects of PRF on soft tissue parameters, including improvements in KMW, PMT, mucosal phenotype, and early healing responses [17, 22–24]. Systematic reviews further suggest that PRF may offer benefits related to postoperative pain reduction, early implant stability, and soft tissue healing, although protocols and outcome measures remain heterogeneous [25].

Importantly, no adverse events or complications were observed during the study period. The absence of reported harms supports the short‐term safety of L‐PRF application in immediate implant placement and is consistent with its autologous and biocompatible nature. However, the limited follow‐up duration does not permit conclusions regarding long‐term safety, tissue stability, or implant survival.

The present findings should be interpreted as short‐term changes in peri‐implant mucosal dimensions rather than evidence of long‐term peri‐implant health benefits. Implant survival, marginal bone loss, peri‐implantitis, mucosal recession, esthetic scores, and patient‐reported outcomes were not assessed. Therefore, the clinical relevance and durability of the observed mucosal changes remain uncertain.

Several limitations should be considered. First, the final follow‐up, performed ~4 months plus 2 weeks after implant placement, reflects only short‐term peri‐implant mucosal changes, and the single‐center design may limit generalizability. Second, blinding the surgeon was not feasible given the nature of the intervention. Third, baseline KMW differed between groups. Although ANCOVA was used to adjust for baseline values, age, and sex, residual influence from baseline soft tissue differences cannot be excluded. Fourth, several socket‐, graft‐, implant system‐, and implant site‐related variables, including socket morphology, buccal bone thickness, gap dimensions, implant position, baseline mucosal phenotype, graft distribution, and the distribution of implant systems between groups, were not consistently available. Fifth, standardized postoperative radiographic outcomes, including marginal bone level changes and peri‐implant bone remodeling, were neither prespecified nor consistently available for all participants. Other postoperative clinical outcomes, including mucosal recession, peri‐implant inflammatory parameters, esthetic outcomes, and patient‐reported outcomes, were also not assessed. Therefore, the observed association between L‐PRF and short‐term peri‐implant mucosal outcomes should be interpreted with caution and should not be considered definitive evidence of an isolated, independent effect of L‐PRF or of long‐term peri‐implant health benefits.

Future studies should include longer follow‐up periods and multicenter designs to evaluate the durability of keratinized tissue gains associated with L‐PRF. Comparative trials assessing L‐PRF against established soft tissue augmentation techniques, such as connective tissue grafts, platelet‐rich plasma, or collagen‐based matrices, are warranted. The inclusion of patient‐reported outcomes and advanced imaging modalities, including cone‐beam computed tomography, may further clarify the clinical relevance of L‐PRF in immediate implant placement.

## 5. Conclusion

Within the limitations of this randomized clinical trial, L‐PRF application was associated with greater short‐term peri‐implant KMW and PMT following immediate implant placement. However, the baseline imbalance in KMW and the absence of consistently recorded socket‐, graft‐, implant system‐, and implant site‐related variables preclude firm attribution of these differences to an isolated, independent effect of L‐PRF. The long‐term stability and clinical relevance of the observed changes remain uncertain because implant survival, marginal bone loss, peri‐implant disease, mucosal recession, esthetic outcomes, and patient‐reported outcomes were not assessed.

## Author Contributions

**Najmehsadat Valedsaravi**: conceptualization, methodology, project administration, supervision, writing – review and editing. **Mehrshad Ahmadi Dizgah**: data curation, investigation, visualization, writing – original draft, writing – review and editing. **Amirhossein Moaddabi**: conceptualization, methodology, supervision, validation, writing – original draft, writing – review and editing. **Abolfazl Hosseinnataj**: data curation, formal analysis, methodology, writing – review and editing.

## Funding

No funding was received for this research.

## Disclosure

All authors have read and approved the final version of the manuscript.

## Conflicts of Interest

The authors declare no conflicts of interest.

## Data Availability

The data supporting the findings of this study are available from the corresponding author upon reasonable request. Access to deidentified participant‐level data is subject to institutional and ethical restrictions related to patient confidentiality.
